# Effect of the peripartum depressive symptoms on the Internet use disorder of their offspring in late childhood: retrospective longitudinal study

**DOI:** 10.1038/s41598-023-50603-5

**Published:** 2024-01-03

**Authors:** Jinhyun Kim, Kyungduk Hurh, Hyunkyu Kim, Eun-Cheol Park, Min Jin Ha

**Affiliations:** 1 Department of Preventive Medicine, Yonsei University College of Medicine, Seoul, Republic of Korea; 2 Institute of Health Services Research, Yonsei University, Seoul, Republic of Korea; 3 Department of Psychiatry, Yonsei University College of Medicine, Seoul, Republic of Korea; 4 Department of Health Informatics and Biostatistics, Graduate School of Public Health, Yonsei University, 50 Yonsei-ro, Seodaemun-gu, Seoul, 03722 Republic of Korea

**Keywords:** Diseases, Medical research, Risk factors, Signs and symptoms

## Abstract

Internet use disorder (IUD) is an emerging social and mental health concern. This study aimed to analyze the relative risk of IUD in late childhood among children whose mothers experienced peripartum depressive symptoms. This study included 762 participants (397 boys and 365 girls) and was conducted in 2017 (aged 9) and 2019 (aged 11). We analyzed the adjusted relative risk of being at high risk for IUD based on whether the mother experienced depressive symptoms during pregnancy or one month after delivery. We also considered the persistence of depressed mood for 4 months after delivery and the severity of peripartum depressive symptoms. From 2017, 20.7% of boys and 14.0% of girls were at high risk of developing IUD. Compared to the non-peripartum depressive group, girls whose mothers experienced peripartum depressive symptoms and those that persisted for 4 months were 1.084 and 1.124 times more likely to be at high risk of IUD (95% confidence interval = 1.005–1.170 and 1.013–1.248), respectively. There were no statistically significant differences among boys. Peripartum depressed mood could be one of risk factors of IUD. IUD needs to be monitored in children whose mothers experienced peripartum depressive symptoms.

## Introduction

Internet use disorder (IUD) is an excessive usage of the Internet characterized by dysfunctional cravings, unregulated duration of usage, and significant psychosocial and functional impairments that are not explained by other disorders^1^. IUD can be classified into two subtypes: predominantly mobile form and predominantly non-mobile form^2^. In this study, we assessed IUD as pathologic online device usage, encompassing both mobile devices and non-mobile devices such as laptops, for various online activities (including all applications and contexts), including online gaming.

IUD in children and adolescents being an emerging health problem due to its high prevalence and adverse effects. Globally, the prevalence of IUD among children and adolescent ranged from 3.2 to 5.4%^3–5^. Based on an annual report on smartphone usage published by the Korean government, the percentage of high-risk smartphone dependence among children rose from 2.3% in 2019 to 4.3% in 2021^6^. There was a research that the prevalence of IUD among Korean children 5–9 years old were 6.1%^7^. Additionally, during the COVID-19 pandemic, the prevalence of IUD is reported to have increased due to pandemic-related distress^8,9^.

However, research on parental psychological and psychiatric conditions, including peripartum depression, associated with IUD, especially in childhood, is limited. While there is research on the positive association between peripartum depression and poor motor, cognitive, emotional, and social development, as well as psychological outcomes, including depression among offsprings^10–18^, these consequences could either act as identified risk factors for IUD or peripartum depression could directly impact the risk of IUD. Moreover, peripartum depression is associated with maternal non-perinatal depression^19–21^, and there is the research about maternal depression could be associated with the risk of IUD^22^. The aim of the study was to investigate the risk of IUD in late childhood among children whose mothers experienced peripartum depressive symptoms, compared with a non-depressive mother group. This investigation extends previous research on the risk of developmental delay or depression in offspring. Due to observed gender differences in the prevalence of pathologic Internet use (14.2% in boys and 10.1% in girls), internet use duration (19.65 h/week in late childhood boys and 16.68 h/week in late childhood girls), and severity of IUD^23–25^, all analyses were conducted with gender stratification. Furthermore, the effect of peripartum depressive symptoms on IUD based on severity and duration was investigated.

## Methods

### Study population and data

**Figure 1:**
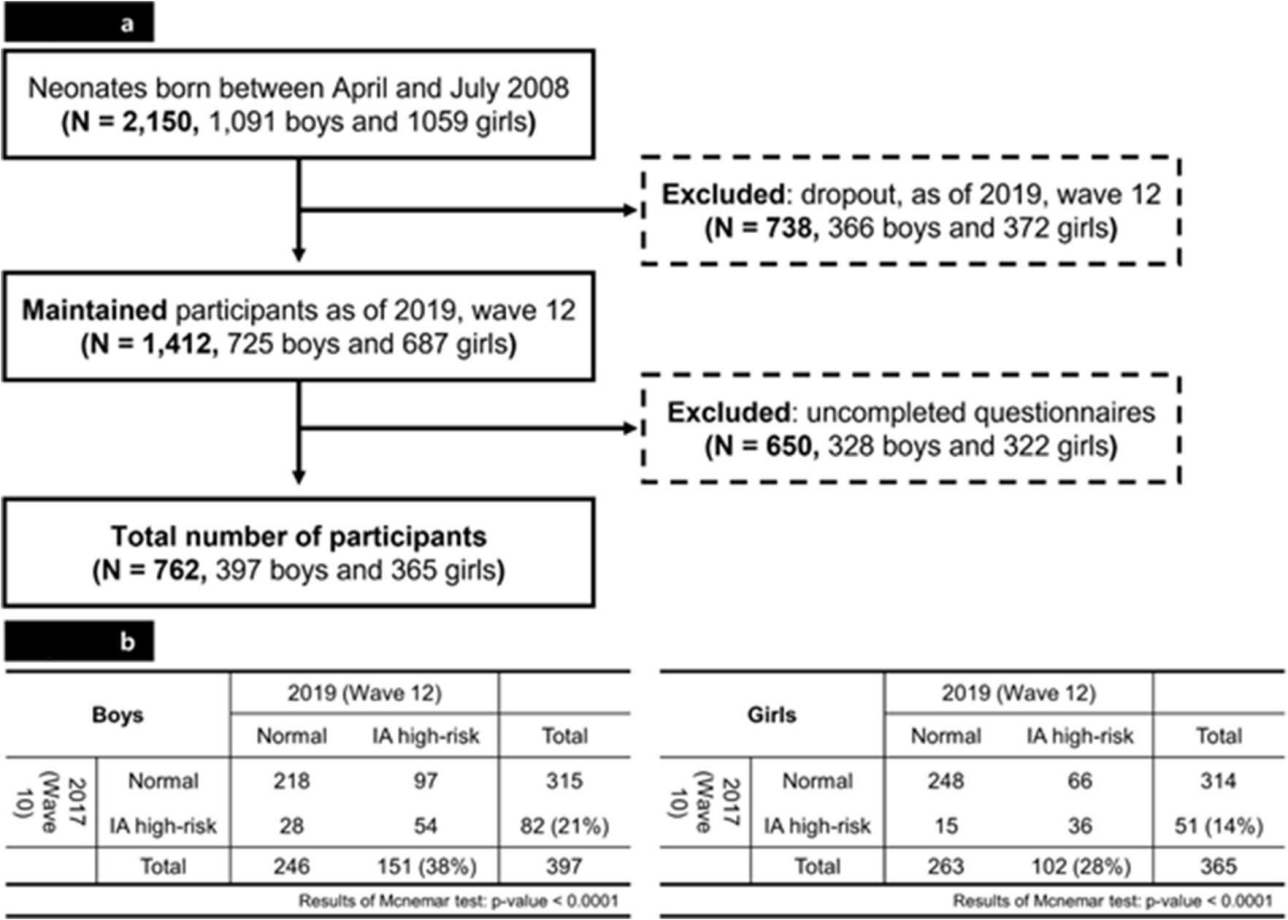
Flow diagram of study participants (**a**) and cross-tables of IUD high-risk in 2017 vs. 2019 for boys and girls (**b**).

Data from the Panel Study on Korean Children (PSKC) Waves 10 (2017, aged 9) and 12 (2019, aged 11), conducted by the Korean Institute of Child Care and Education, were analyzed in this study. Wave selection was decided based on data availability. Survey items for IUD evaluation were initiated in 2017, and major study variables, such as the Korean version of the Child Behaviour Checklist (K-CBCL) which was the caregiver rating screening tool for children’s behavioural problems, were not measured in 2018. The PSKC is a nationwide, annually conducted panel study since 2008, when the participants were newborns, and will last until 2027 when they reach the age of 19 years. PSKC collected data on newborn participants and their mothers, fathers, and primary teachers through self-reporting. Multistratified sampling based on provinces and medical institutes was used in this study. A total of 2150 neonates born between April and July 2008 were included in this study. The exclusion criteria were poor health status of the newborn or postpartum mother, poor Korean language skills of the mother and father, and multiple births. The socioeconomic and health-related characteristics of children and their parents were investigated in the PSKC to track the growth processes of Korean children. The investigation was conducted through household visits conducted by trained interviewers. The survey did not include a 'prefer not to answer' option for any of the questions. The baseline number of panel participants was 2150 (in 2008), and the panel retention rates were 71.4% (n = 1484) in 2017 and 67.9% (n = 1412) in 2019. A total of 722 participants in 2017 and 650 in 2019 who did not respond to at least one of the study variables were excluded. Our study was based on the measurements of 762 participants (Fig. 1a).

### Measures

#### Peripartum depressive symptoms

Maternal depressive symptoms were evaluated three times: immediately after delivery, one month after delivery, and four months after delivery in 2008, based on the Korean version of the Kessler 6-item Psychological Distress Scale (K6 scale)^26,27^. Depressive symptoms were evaluated by asking participants’ mothers about feelings of nervousness, loss of energy, restlessness, fatigue, worthlessness, and sadness in the past month; participants answered on a 5-point Likert scale. The cutoff value for depressive symptoms was 14 points out of the total sum of scores of 30 points (i.e. the depressive range was 14–30). The severity of depressive symptoms was determined by further classifying the depressive range into 14–18 for ‘mild’ and 19–30 for ‘severe’ depressive symptoms. Based on Diagnostic and statistical manual of mental disorders (DSM), peripartum depression is defined as depressive symptoms during pregnancy or within a month of delivery^28^. Therefore, in this study, we defined peripartum depression as depressive symptoms present in both the surveys immediately after delivery and one month after delivery. For subgroup analyses, persistent peripartum depressive symptoms were defined as the continuation of mild or severe depressive symptoms until four months after delivery. The percentage of peripartum depressive symptoms among the mothers of the study participants was 30.97% (25.07% with mild symptoms and 5.90% with severe symptoms) and 14.70% of the mothers experienced persistent peripartum depressive symptoms (47.46% of mothers with peripartum depressive symptoms).

#### Internet use disorder and Internet usage duration

A 15-item Internet Addiction Proneness Scale for Youth (KS-II) developed by the National Information Society Agency was used for Internet addiction evaluation^29^. The KS-II was used in 2017 and 2019. Usage of the Internet contains every online activities including gaming, using social network service platforms, watching videos.

The KS-II relies on the parents’ rating scale due to the young age of the participants and consists of four major fields: adaptive function disturbance (five items), virtual life orientation (two items), withdrawal (four items), and tolerance (four items). For instance, “He/she is on the Internet all night for one or more days”, “When not on the Internet, he/she cannot concentrate on other tasks and looks anxious” or “His/her Internet time is increasing”. A total score of 4-point Likert scale for all items and each major field were considered for Internet addiction screening. The participants were divided into two groups: a high-risk IUD group and a normal group. There were two diagnostic criteria for high-risk IUD based on the KS-II. First, a total score of 30 points or more was classified as the high-risk IUD group. Alternatively, 14 or more points for adaptive function disturbance, 12 or more for withdrawal, and 11 or more for tolerance were classified in the high-risk IUD group.

### Covariates

Time-varying child and parent/household variables were also included as covariates. The child variables were age (9- or 11-year-old), having a smartphone, feeling of happiness, sleep duration, subjective health status, and K-CBCL. Participants were categorized into two age groups, 9 and 11, based on their age during both PSKC wave 10 and wave 12, when they were elementary school students in Korea. Having a smartphone was classified into two groups depending on whether the child owned a smartphone. Sleep duration was divided into two groups based on the recommended 9-h duration for school-aged children^30^. Subjective health status was divided into three groups: low, middle, and high. Feeling of happiness was evaluated using self-reported happiness with life, which is part of the UK Millennium Cohort Study Child Paper Self Completion Questionnaire^31^. Caregiver-rated behavior problems were evaluated using K-CBCL^32^. A T-score higher than 64 (95 percentile), either internalizing or externalizing, was adopted as the criterion for behavioral problems^33^. All variables except ‘feeling of happiness’ were reported by parents. All child variables, with the exception of K-CBCL, were queried directly to the children (participants), while K-CBCL was directed to their parents.

The variables for parents/households were family composition, economic and employment status, and the current depressed mood of each parent. Family composition was divided into two groups: living with both parents and living with others. Economic status was divided into three categories based on the income quintile of Korea in the quarter of the year when the PSKC was implemented. Employment status was classified into three categories: both parents were employed, one parent was employed, and both parents were unemployed. The depressed mood of each parent was evaluated using the K6 scale, asking them about feelings of nervousness, loss of energy, restlessness, fatigue, worthlessness, and sadness in the past month, and participants with scores in the range of 14–30 were classified into the depressive group. All parent/household variables were directed to the participants' parents.

### Statistical analysis

All analyses were conducted stratified by gender. McNemar’s test was used to assess the difference between the probabilities of high-risk IUD in 2017 and 2019. A chi-square test was performed to determine whether there was a significant association between high-risk IUD and each of the socioeconomic and health-related characteristics of children and their parents at a specific time. A generalized estimating equation Poisson regression model with a log link function was used for the longitudinal data (2017, 2019) and an autoregressive (AR) working correlation matrix, which showed lower QIC statistics than the independent working correlation matrix, was applied. In the comprehensive analyses, we adjusted for potential covariates and various child-related variables in the Poisson regression model. Subgroup analyses were conducted to analyze the detailed effects of persistent peripartum depressive symptoms and covariates. The results were expressed as adjusted relative risk (aRR) with 95% confidence intervals (CI). The weight variables provided by the PSKC for the weighted regression were included in the analysis. The variance inflation factors for all variables were checked in each regression model to be less than 1.2, so there was no evidence of multicollinearity. SAS software version 9.4 (SAS Institute, Cary, North Carolina, USA) was used for all statistical analyses. Statistical significance was set at p < 0.05.

### Ethics approval and consent to participate

This study was conducted in accordance with the Declaration of Helsinki. This study was approved by the Institutional Review Board of the Korea Institute of Child Care and Education. All participants were informed about the study and provided informed consent. Parental consent was obtained from all participants because they were younger than 18 years.

## Results

A total of 762 participants were included, contributing 1524 observations (Fig. 1a). In the cohort of 2150 participants, 468 participants' mothers did not provide responses to peripartum depression questions, 842 participants did not respond to IUD questions, and 1058 participants and their parents did not answer questions regarding covariates during the survey. The results of McNemar’s test are shown in Fig. 1b. For boys (n = 397), the marginal probability of high-risk IUD increased from 0.21 in 2017 to 0.38 in 2019 (p < 0.0001), while for girls (n = 365), the probability increased from 0.14 in 2017 to 0.28 in 2019 (p < 0.0001). For 2017 (Wave 10), chi-square tests for the general characteristics of the study population according to gender and high-risk IUD are shown in Table 1. Girls whose mothers experienced peripartum depressive symptoms had a significantly higher percentage of high-risk IUD (24.3%) than the control group (9.7%), but there was no significant difference among boys. Meanwhile, there was significantly higher high-risk IUD percentage for the existence of caregiver-rated behaviour problems and current depressive symptoms in mothers in both genders.

**Table 1:** Results of chi-square test of socioeconomic and health-related characteristics of the study population from 2017.

Variables	Boy (N = 397, 52.1%)				p-value	Girl (N = 365, 47.9%)				p-value
	Normal		IUD high-risk			Normal		IUD high-risk		
	N	(%)	N	(%)		N	(%)	N	(%)	
Total	315	(79.3)	82	(20.7)		314	(86.0)	51	(14.0)	
Peripartum depressive symptoms					0.2491					**0.0045**
None	220	(82.1)	48	(17.9)		233	(90.3)	25	(9.7)	
Depressive	95	(73.6)	34	(26.4)		81	(75.7)	26	(24.3)	
Severity subgroup					0.1661					**0.0025**
None	220	(82.1)	48	(17.9)		233	(90.3)	25	(9.7)	
Mild depressive symptoms	76	(76.0)	24	(24.0)		67	(73.6)	24	(26.4)	
Severe depressive symptoms	19	(65.5)	10	(34.5)		14	(87.5)	2	(12.5)	
Persisted symptoms subgroups					0.2913					**0.0045**
None	220	(82.1)	48	(17.9)		233	(90.3)	25	(9.7)	
Without persistence	47	(70.1)	20	(29.9)		47	(82.5)	10	(17.5)	
With persistence	48	(77.4)	14	(22.6)		34	(68.0)	16	(32.0)	
Having smartphone					0.8523					0.3504
No	131	(80.9)	31	(19.1)		75	(86.2)	12	(13.8)	
Yes	184	(78.3)	51	(21.7)		239	(86.0)	39	(14.0)	
Happiness					0.2758					0.7352
Happy	278	(80.1)	69	(19.9)		288	(85.7)	48	(14.3)	
Unhappy	37	(74.0)	13	(26.0)		26	(89.7)	3	(10.3)	
Sleep duration					0.2076					**0.0315**
Less than 9-h	64	(71.9)	25	(28.1)		54	(88.5)	7	(11.5)	
More than 9-h	251	(81.5)	57	(18.5)		260	(85.5)	44	(14.5)	
Subjective health status					0.5521					0.4998
Low	41	(80.4)	10	(19.6)		26	(83.9)	5	(16.1)	
High	274	(79.2)	72	(20.8)		288	(86.2)	46	(13.8)	
Caregiver rating behavior problems					**0.0019**					**0.0431**
No	287	(82.0)	63	(18.0)		298	(87.1)	44	(12.9)	
Yes	28	(59.6)	19	(40.4)		16	(69.6)	7	(30.4)	
Family composition					0.1572					0.7791
Living with both parents	293	(78.6)	80	(21.4)		298	(86.4)	47	(13.6)	
The others	22	(91.7)	2	(8.3)		16	(80.0)	4	(20.0)	
Economic status					0.0519					0.3845
Low	35	(76.1)	11	(23.9)		46	(80.7)	11	(19.3)	
Middle	190	(77.9)	54	(22.1)		163	(85.3)	28	(14.7)	
High	90	(84.1)	17	(15.9)		105	(89.7)	12	(10.3)	
Employment status (parents)					0.3541					0.3967
Both not employed	11	(68.8)	5	(31.3)		9	(75.0)	3	(25.0)	
Employed (one of parents)	156	(82.5)	33	(17.5)		150	(88.2)	20	(11.8)	
Both employed	148	(77.1)	44	(22.9)		155	(84.7)	28	(15.3)	
Current depressed mood (mother)					** < 0.0001**					**0.0082**
No	235	(85.1)	41	(14.9)		232	(88.5)	30	(11.5)	
Yes	80	(66.1)	41	(33.9)		82	(79.6)	21	(20.4)	
Current depressed mood (father)					**0.0377**					0.5473
No	223	(82.9)	46	(17.1)		214	(85.6)	36	(14.4)	
Yes	92	(71.9)	36	(28.1)		100	(87.0)	15	(13.0)	

Significant values are in bold.

**Figure 2:**
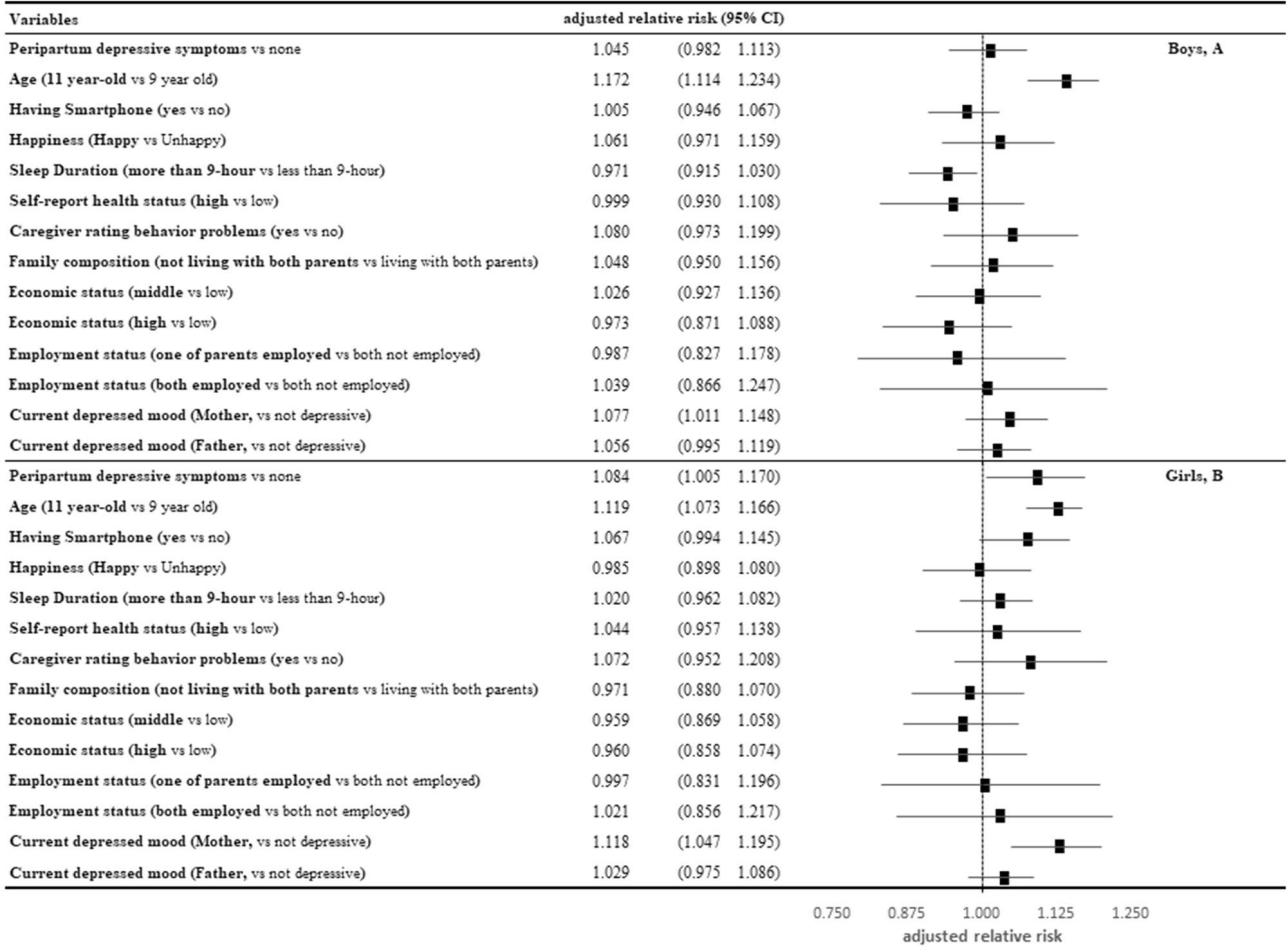
Relationship between peripartum depressive symptoms and high risk of Internet addiction, adjusted for covariates for boys (**A**) and girls (**B**).

Using the longitudinal features measured in 2017 and 2019, we investigated the risk factors for high-risk IUD, including peripartum depressive symptoms (Fig. 2). Compared to the non-peripartum depressive group, girls whose mothers experienced postpartum depressive symptoms were 1.084 times more likely to be at high risk for IUD (aRR: 1.084, 95% CI 1.005–1.169) but no statistical significance in boys (aRR: 1.045, 95% CI 0.981–1.113). Meanwhile, eleven-year-old children were more likely to be at high risk for IUD than 9-year-old children. Children whose mothers were currently depressive were more likely to be at high risk for IUD in both genders compared to the non-depressive mother group.

**Figure 3:**
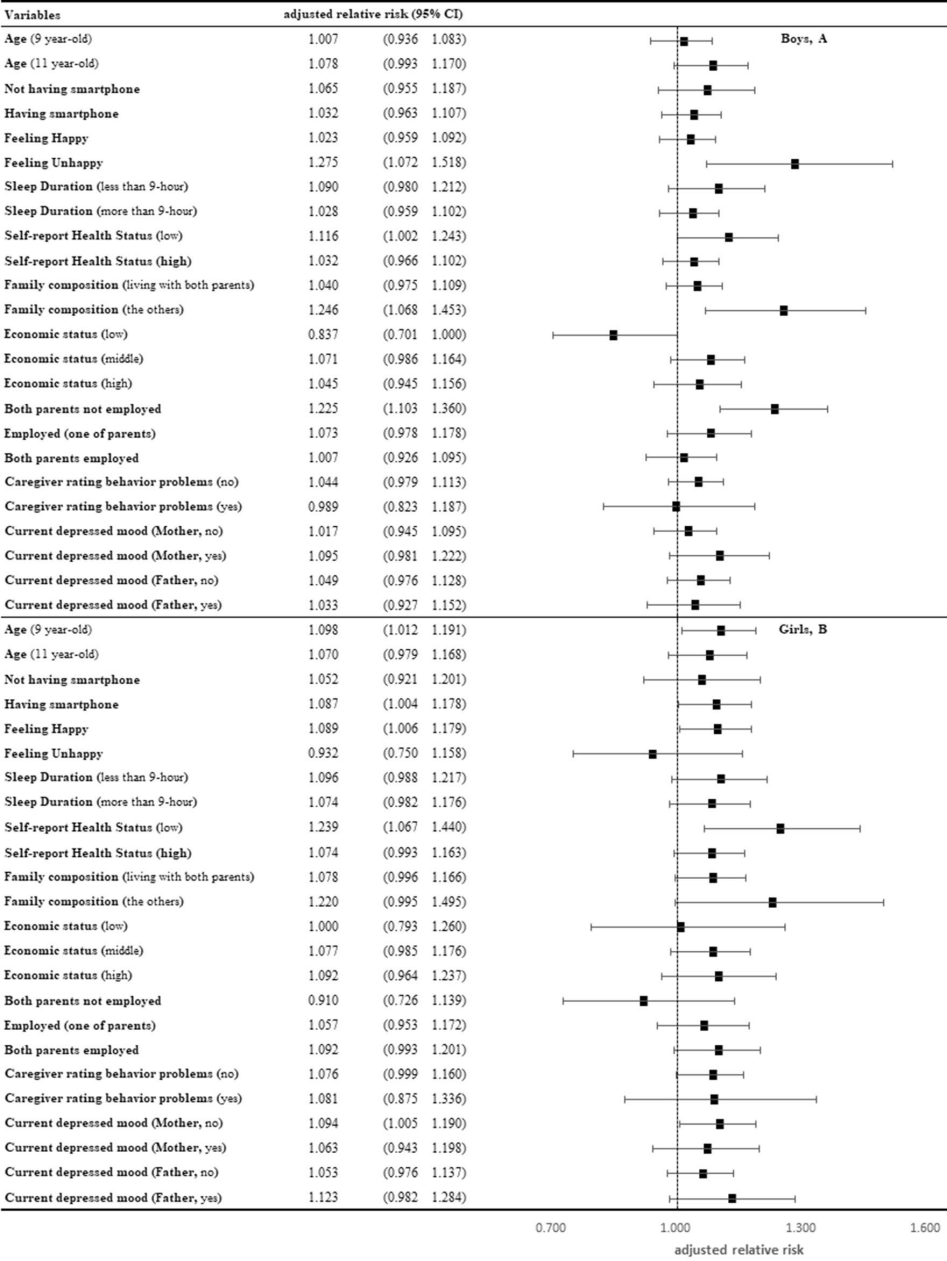
Subgroup analyses based on several socioeconomic and health related factors for boys (**A**) and girls (**B**).

In each subgroup, the aRR of high-risk patients with IUD was estimated according to socioeconomic and health-related factors (Fig. 3). After other covariates were adjusted, boys who were unhappy, had low self-reported health status, were not living with either parent, or whose parents were unemployed were statistically significant. Girls who had a smartphone, were happy, and had low self-reported health status and mothers who are currently depressed were statistically significant.

**Figure 4:**
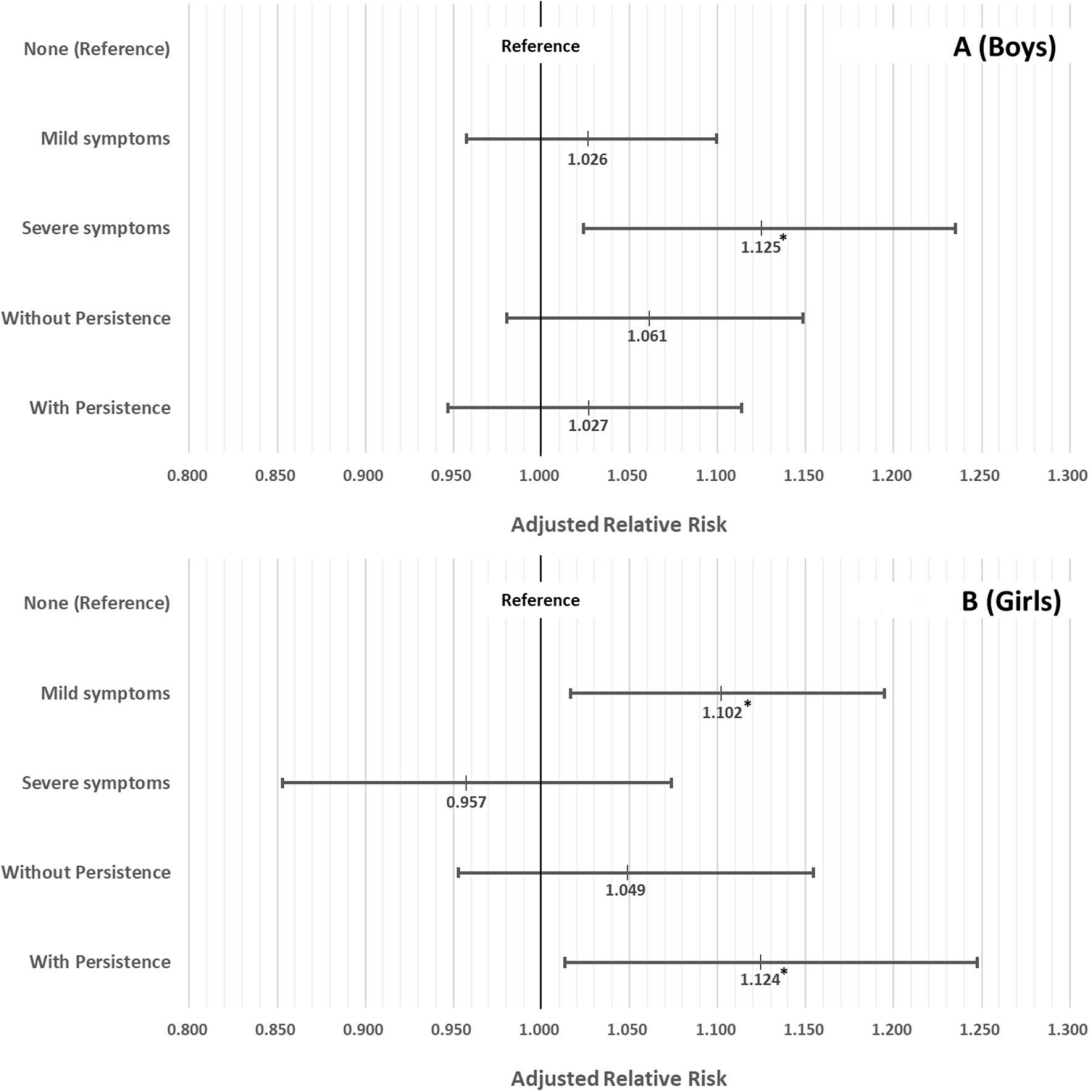
Subgroup analyses based on severity and 4-month persistence of peripartum depressive symptoms with covariates adjustment among boys (**A**) and girls (**B**). *Statistically significant. Adjusted for age, smartphone use, happiness, sleep duration, subjective health status, caregiver rating behavioral problems, family composition, parental economic status, parental employment status, and current depressed mood of parents.

Figure 4 shows the results of the subgroup analyses based on the severity and persistence of peripartum depressive symptoms. Boys whose mothers experienced severe peripartum depressive symptoms were 1.125 times more prone to high-risk IUD than those in the no peripartum symptoms group (aRR: 1.125, 95% CI 1.024–1.235). Girls whose mothers experienced mild peripartum depressive symptoms were 1.102 times more likely to be at high risk for IUD (aRR: 1.102, 95% CI 1.016–1.195). In addition, girls whose mothers experienced 4-month persisted peripartum depression were 1.124 times more at risk for high-risk IUD than those of the reference group (aRR: 1.124, 95% CI 1.013–1.248).

## Discussion

In our study, peripartum depressive symptoms in mothers posed a greater risk for IUD among their daughters. Understanding the neurobehavioral risk factors and pathophysiology of IUD is crucial for developing effective prevention and treatment strategies. According to the cognitive behavior theory, cognitive distortions, including rumination and generalization, are a central cause of pathologic Internet use^34^. Additionally, cultural factors, such as Internet accessibility, have also been recognized as risk factors^35^. There are also potential neurobiological factors, such as immature prefrontal cortex, anterior/posterior cingulate cortex, dopamine transporter expression levels^36–40^, and structural changes in the brain due to IUD was also found^41^. Based on sociodemographic and health-related factors, male sex and mental disorder morbidity are associated with IUD^42^. Drinking behavior and recent stressful events may be risk factors for IUD^43^. Additionally, there is a positive association between IUD and various psychiatric disorders and symptoms, including anxiety, depression, impaired daily function, eating disorder, sleep problems, non-suicidal self-injury (NSSI), and suicidality among children and adolescents^7,44–47^. Substance addiction (cannabis, sedatives, stimulants, alcohol) and behavioral addictions (gambling) were associated with IUD^48^.

Based on the findings of these studies, a possible explanation for the results is that poor outcomes due to peripartum depression, such as offspring's depression or poor development, could increase the risk of IUD^45,49^. Additionally, it is possible that peripartum depression and IUD share the same risk factors, including genetic predisposition or low social support, marital/familial difficulties^50–53^. Meanwhile, IUD could be an independent clinical behavioral phenotype among distressed children, because there was statistical significance irrespective of behavioral problems which were evaluated by CBCL in the study (Fig. 3). Although further research on the underlying mechanism and cause-effect relationship is needed, peripartum depression could be a possible predictive factor for IUD in offspring and provide clues for the pathophysiology of IUD. Given the significant association, it is essential to consider screening offspring of mothers who have experienced peripartum depression for IUD. Additionally, further research is needed to determine whether IUD could be considered one of the phenotypes of poor mental health or an independent diagnosis among children.

According to the results of subgroup analyses (Fig. 3A,B), it appears that peripartum depression may have different effects based on gender. Boys, particularly those under adverse environmental or psychological conditions (such as not living with parents, having both parents without employment, and feeling unhappy), were more likely to be at high risk for IUD when their mothers had experienced peripartum depression, whereas this pattern was not observed among girls. Therefore, while girls, in general, were more susceptible to the impact of peripartum depression, boys, especially under adverse conditions, showed a heightened vulnerability. The discovery that boys, whose mothers had experienced severe peripartum depressive symptoms, were at a higher risk of IUD supports the notion that boys may be less vulnerable to IUD due to peripartum depression (Fig. 4). This gender disparity could be attributed to the closer relationship and emotional sharing typically observed between daughters and mothers, as reflected in our results^54–56^.

The study represents the first investigation into the association between peripartum depression and IUD risk in offspring, utilizing longitudinal follow-up data. However, this study has several limitations. However, this study had several limitations. First, peripartum depression was not clinically evaluated using an official diagnostic approach or a common scale for peripartum depression, such as the Edinburgh depression scale^57^. In addition, because the data were obtained through self-reports and parent ratings, their accuracy cannot be guaranteed. Also, there are other potential covariates such as IUD among parents, peripartum depression among fathers, treatment of postpartum depression, or other psychiatric disorders. Moreover, because the participants were all Koreans and the study was conducted in Korea, the results cannot be generalized to other countries or ethnicities with different social cultures. Furthermore, there was a long period between the delivery and time of IUD assessment; further, there was an indirect relationship between parents and offspring. Several factors influenced the results in individual, familial, and social aspects, including the rapidly changing technology and environment of the Internet.

In conclusion, an association between peripartum depression in mothers and IUD in their offspring was observed in this study. Specifically, daughters of mothers with at least 4-month of persistent peripartum depression were at risk of IUD. Several psychiatric conditions, including IUD, need to be followed up on in children whose mothers experienced postpartum depression, and the underlying mechanisms for the onset of IUD need to be investigated.

## Data Availability

The data analyzed in this study were obtained from the Panel Study of Korean Children, which is publicly available. All data can be obtained from the official website: (https://panel.kicce.re.kr/pskc/index.do).

## References

[1] Kuss DJ, Pontes HM (2018). Internet Addiction.

[2] Montag C, Wegmann E, Sariyska R, Demetrovics Z, Brand M (2021). How to overcome taxonomical problems in the study of Internet use disorders and what to do with “smartphone addiction”?. J. Behav. Addict..

[3] Rikkers W, Lawrence D, Hafekost J, Zubrick SR (2016). Internet use and electronic gaming by children and adolescents with emotional and behavioural problems in Australia - results from the second Child and Adolescent Survey of Mental Health and Wellbeing. BMC Public Health.

[4] Wartberg L, Kriston L, Kammerl R, Petersen KU, Thomasius R (2015). Prevalence of pathological internet use in a representative German sample of adolescents: Results of a latent profile analysis. Psychopathology.

[5] Takahashi M (2018). Prevalence of pathological and maladaptive Internet use and the association with depression and health-related quality of life in Japanese elementary and junior high school-aged children. Soc. Psychiatry Psychiatr. Epidemiol..

[6] Ministry of Science and ICT & National Information Society Agency 2021 The survey on smart phone overdependence. https://www.nia.or.kr/site/nia_kor/ex/bbs/View.do?cbIdx=65914&bcIdx=24288&parentSeq=24288 (2022).

[7] Lim Y, Nam SJ (2020). Exploring factors related to problematic internet use in childhood and adolescence. Int. J. Ment. Health Addict..

[8] Lee JJ, Shin SH (2022). Associations between fear of COVID-19, depression, and internet addiction in South Korean adults. Healthcare (Basel).

[9] Li YY (2021). Internet addiction increases in the general population during COVID-19: Evidence from China. Am. J. Addict..

[10] Talge NM (2007). Antenatal maternal stress and long-term effects on child neurodevelopment: How and why?. J. Child Psychol. Psychiatry.

[11] Van den Bergh BR, Mulder EJ, Mennes M, Glover V (2005). Antenatal maternal anxiety and stress and the neurobehavioural development of the fetus and child: Links and possible mechanisms. A review. Neurosci. Biobehav. Rev..

[12] Zietlow AL (2022). Study protocol of the COMPARE-Interaction study: The impact of maternal comorbid depression and anxiety disorders in the peripartum period on child development. BMJ Open.

[13] Davalos DB, Yadon CA, Tregellas HC (2012). Untreated prenatal maternal depression and the potential risks to offspring: A review. Arch. Womens Ment. Health.

[14] Letourneau NL (2012). Postpartum depression is a family affair: Addressing the impact on mothers, fathers, and children. Issues Ment. Health Nurs..

[15] Vänskä M (2011). Maternal pre-and postnatal mental health trajectories and child mental health and development: Prospective study in a normative and formerly infertile sample. Int. J. Behav. Dev..

[16] Quevedo LA (2012). The impact of maternal post-partum depression on the language development of children at 12 months. Child Care Health Dev..

[17] Koutra K (2013). Antenatal and postnatal maternal mental health as determinants of infant neurodevelopment at 18 months of age in a mother-child cohort (Rhea Study) in Crete, Greece. Soc. Psychiatry Psychiatr. Epidemiol..

[18] Oyetunji A, Chandra P (2020). Postpartum stress and infant outcome: A review of current literature. Psychiatry Res..

[19] Goodman JH (2004). Postpartum depression beyond the early postpartum period. J. Obstet. Gynecol. Neonatal Nurs..

[20] Kettunen P, Koistinen E, Hintikka J (2014). Is postpartum depression a homogenous disorder: Time of onset, severity, symptoms and hopelessness in relation to the course of depression. BMC Pregnancy Childbirth.

[21] Dekel S (2019). The dynamic course of peripartum depression across pregnancy and childbirth. J. Psychiatr. Res..

[22] Oh Y, Kim H, Joung YS (2021). Problematic internet use in children according to maternal depression trajectories: A population-based cohort study with 9-year follow-up. J. Psychiatr. Res..

[23] Vigna-Taglianti F (2017). Problematic Internet use among high school students: Prevalence, associated factors and gender differences. Psychiatry Res..

[24] Dufour M (2016). Gender difference in internet use and internet problems among Quebec high school students. Can. J. Psychiatry Revue Can. Psychiatrie.

[25] Baloglu M, Sahin R, Arpaci I (2020). A review of recent research in problematic internet use: Gender and cultural differences. Curr. Opin. Psychol..

[26] Shin N (2007). Panel Study on Korean Children: Preliminary Report.

[27] Kessler RC (2002). Short screening scales to monitor population prevalences and trends in non-specific psychological distress. Psychol. Med..

[28] American Psychiatric Association (2013). Diagnostic and Statistical Manual of Mental Disorders (DSM-5®).

[29] Sin G, Kim D, Jeung Y (2011). Third Standardization of Korean Internet Addiction Proneness Scale.

[30] Hirshkowitz M (2015). National sleep foundation’s updated sleep duration recommendations: Final report. Sleep Health.

[31] Connelly R, Platt L (2014). Cohort profile: UK Millennium cohort study (MCS). Int. J. Epidemiol..

[32] Oh K, Lee H, Hong K, Ha E (1997). Korean Version of the Child Behavior Checklist (K-CBCL).

[33] Achenbach TM (1991). Manual for the Child Behavior Checklist/4-18 and 1991 Profile.

[34] Davis RA (2001). A cognitive-behavioral model of pathological Internet use. Comput. Human Behav..

[35] Shaw M, Black DW (2008). Internet addiction: Definition, assessment, epidemiology and clinical management. CNS Drugs.

[36] Hou H (2012). Reduced striatal dopamine transporters in people with internet addiction disorder. J. Biomed. Biotechnol..

[37] Meng Y, Deng W, Wang H, Guo W, Li T (2015). The prefrontal dysfunction in individuals with Internet gaming disorder: A meta-analysis of functional magnetic resonance imaging studies. Addict. Biol..

[38] Yuan K (2013). Cortical thickness abnormalities in late adolescence with online gaming addiction. PLoS One.

[39] Dong G, Devito EE, Du X, Cui Z (2012). Impaired inhibitory control in 'internet addiction disorder': A functional magnetic resonance imaging study. Psychiatry Res..

[40] Caballero A, Tseng KY (2016). GABAergic function as a limiting factor for prefrontal maturation during adolescence. Trends Neurosci..

[41] Vercillo K (2020). Internet Addiction.

[42] Tsai HF (2009). The risk factors of Internet addiction–a survey of university freshmen. Psychiatry Res..

[43] Lam LT, Peng ZW, Mai JC, Jing J (2009). Factors associated with Internet addiction among adolescents. Cyberpsychol. Behav..

[44] Kim H (2023). Suicide and non-suicidal self-injury from internet addiction among Korean adolescents. J. Korean Acad. Child Adolesc. Psychiatry.

[45] El Asam A, Samara M, Terry P (2019). Problematic internet use and mental health among British children and adolescents. Addict. Behav..

[46] Alimoradi Z (2019). Internet addiction and sleep problems: A systematic review and meta-analysis. Sleep Med. Rev..

[47] Hinojo-Lucena FJ, Aznar-Diaz I, Caceres-Reche MP, Trujillo-Torres JM, Romero-Rodriguez JM (2019). Problematic Internet use as a predictor of eating disorders in students: A systematic review and meta-analysis study. Nutrients.

[48] Sela Y, Bar-Or RL, Kor A, Lev-Ran S (2021). The Internet addiction test: Psychometric properties, socio-demographic risk factors and addictive co-morbidities in a large adult sample. Addict. Behav..

[49] Park MH (2011). Preliminary study of Internet addiction and cognitive function in adolescents based on IQ tests. Psychiatry Res..

[50] Howard LM (2014). Non-psychotic mental disorders in the perinatal period. Lancet.

[51] Norhayati MN, Hazlina NH, Asrenee AR, Emilin WM (2015). Magnitude and risk factors for postpartum symptoms: A literature review. J. Affect. Disord..

[52] Viktorin A (2016). Heritability of perinatal depression and genetic overlap with nonperinatal depression. Am. J. Psychiatry.

[53] Couto TC (2015). Postpartum depression: A systematic review of the genetics involved. World J. Psychiatry.

[54] Fivush R, Brotman MA, Buckner JP, Goodman SH (2000). Gender differences in parent–child emotion narratives. Sex Roles.

[55] Kerig PK, Cowan PA, Cowan CP (1993). Marital quality and gender differences in parent-child interaction. Dev. Psychol..

[56] Starrels ME (1994). Gender differences in parent-child relations. J. Fam. Issues.

[57] Murray L, Carothers AD (1990). The validation of the Edinburgh post-natal depression scale on a community sample. Br. J. Psychiatry.

